# Prognostic impact of shift to low visceral fat mass after neoadjuvant chemotherapy in patients with esophageal cancer

**DOI:** 10.1002/cnr2.2084

**Published:** 2024-08-19

**Authors:** Sachiyo Onishi, Masahiro Tajika, Tsutomu Tanaka, Keisaku Yamada, Yoshitaka Inaba, Tetsuya Abe, Kei Muro, Masahito Shimizu, Yasumasa Niwa

**Affiliations:** ^1^ Department of Endoscopy Aichi Cancer Center Hospital Nagoya Japan; ^2^ Department of Diagnostic and Interventional Radiology Aichi Cancer Center Hospital Nagoya Japan; ^3^ Department of Gastroenterological Surgery Aichi Cancer Center Hospital Nagoya Japan; ^4^ Department of Clinical Oncology Aichi Cancer Center Hospital Nagoya Japan; ^5^ Department of Gastroenterology/Internal Medicine Gifu University Graduate School of Medicine Gifu Japan

**Keywords:** esophageal cancer, neoadjuvant chemotherapy, sarcopenia, shift to low visceral fat mass

## Abstract

**Background:**

Based on the JCOG1109 trial, it is suggested that the combination of docetaxel, cisplatin, and 5‐fluorouracil (DCF) could potentially become a standard neoadjuvant chemotherapy regimen, alongside the conventional 5‐fluorouracil and cisplatin (CF) therapy, for esophageal cancer. However, there are few reports on the impact of body composition changes associated with neoadjuvant chemotherapy on prognosis.

**Aim:**

Our study aimed to explore the effect of different neoadjuvant chemotherapy regimens on body composition during treatment and the impacts of body composition changes on their prognosis.

**Methods and results:**

This is a retrospective study of 215 patients with advanced thoracic esophageal cancer who had surgery after neoadjuvant chemotherapy from 2013 to 2019. Computed tomography scans were performed before and after neoadjuvant chemotherapy to assess body composition. Skeletal muscle mass index (SMI) was calculated by dividing total skeletal muscle mass at the 3rd lumbar level by the square of height, while visceral and subcutaneous fat masses were measured at the level of umbilicus. Patients in the lowest 25% of both sexes were classified into the low visceral fat and low subcutaneous fat groups, respectively. Of the patients enrolled, 178 were male and 37 were female. Among them, 91 had clinical Stage II disease, and 124 had clinical Stage III disease. Additionally, 146 patients received neoadjuvant chemotherapy CF, and 69 received neoadjuvant chemotherapy DCF. Comparing the DCF and CF groups, the DCF group consisted of significantly younger patients (*p* < .01), a higher proportion of males (*p* = .03), and a greater number of clinical Stage III cases (*p* < .01). However, although percent change in SMI and visceral fat mass was not significantly different between two regimens, percent change in subcutaneous fat mass was significant in the DCF group. The major prognostic factors for patients undergoing surgery after neoadjuvant chemotherapy for thoracic esophageal cancer were clinical Stage III, transition to low visceral fat, and response rating (SD/PD), while the specific neoadjuvant chemotherapy regimen did not significantly influence the outcomes.

**Conclusion:**

This study suggests that prevention of the shift to low visceral fat throughout the neoadjuvant chemotherapy process should improve patient outcomes.

## 1 INTRODUCTION

The Guidelines for the Diagnosis and Treatment of Esophageal Carcinoma^1^ have endorsed the application of manageable neoadjuvant chemotherapy with definitive surgery for advanced Stage II and III esophageal cancer. Traditionally, neoadjuvant chemotherapy with cisplatin + 5‐fluorouracil (5‐FU) (CF) has served as the standard therapy for this cancer type, based on findings from the Japan Clinical Oncology Group (JCOG) 9907 trial.^2^ Recently, a phase III randomized trial, JCOG1109, evaluated the effectiveness of CF compared to 5‐FU + docetaxel (DOC) + cisplatin (DCF) and neoadjuvant chemoradiotherapy (CRT) in advanced Stage II/III esophageal cancer.^3^ The results showed that CRT did not show meaningful superiority over CF in overall survival (OS), while DCF significantly improved OS compared to CF. Consequently, in Japan, with the revised the Guidelines for the Diagnosis and Treatment of Esophageal Carcinoma, DCF will become another standard therapy for Stage II/III esophageal cancer.

Despite its high responder rate^4^ and improved in OS and progression‐free survival,^5^ DCF treatment often results in treatment‐related adverse events, notably gastrointestinal toxicities.^6^ DCF regimens as modified have been employed to mitigate hematologic toxicity in gastric cancer, but oral mucosal disorders remain prevalent.^7^ Chemotherapy‐induced gastrointestinal toxicity can adversely affect the nutritional status of the patient, potentially resulting in both discontinuation of chemotherapy and a decreased quality of life associated with treatment. Several reports recommend the use of enteral nutrition (EN) as a form of supportive care therapy to manage such treatment‐related toxicities.^8^ However, research on managing nutrition for patients undergoing DCF treatment, renowned for its considerable toxicity to the digestive organs, remains limited.

Our previous study emphasized prognostically importance of changes in body composition under neoadjuvant chemotherapy. This study defines sarcopenia as a loss of skeletal muscle mass, post‐neoadjuvant chemotherapy sarcopenia is increasingly recognized as graver prognostic factor than pre‐neoadjuvant chemotherapy sarcopenia,^9^ and it may be exacerbated by therapy related digestive tract toxicity, along with the cachexia commonly linked to malignancy. We postulated that the various changes in body composition that may occur during neoadjuvant chemotherapy could impact prognosis.

The purpose of our study was to compare body composition and changes in body composition between conventional CF therapy and DCF therapy, which will be used more frequently in the future as an alternative to conventional CF therapy. Furthermore, we will clarify the prognostic factors for patients who have undergone neoadjuvant chemotherapy and examine the influence of changes in body composition on prognosis.

## 2 METHODS

### 2.1 Study design and patients

This study is a retrospective cohort study. Between April 2013 and December 2019, 215 individuals diagnosed with clinical Stage II/III esophageal squamous cell carcinoma and receiving treatment at the Aichi Cancer Center underwent comprehensive CT scans and esophagogastroduodenoscopy (EGD) pre‐ and post‐receiving neoadjuvant chemotherapy. Some cases overlap with the patients included in our previous study.^9^

### 2.2 Ethical considerations

This study obtained approval from the Ethics Committee of Aichi Cancer Center Hospital. The Aichi Cancer Center Ethics Review Committee approved under Research No. IR031183. In addition, this study was carried out in compliance with Declaration of Helsinki, as update in 1983.

### 2.3 Neoadjuvant chemotherapy

A collaborative approach was adopted by the Department of Digestive Surgery, Endoscopy, Radiation Oncology, and Clinical Oncology to assess stage and formulate treatment strategies. The choice of chemotherapy regimen was deliberated and determined during joint meetings. The CF therapy was administered in 2 cycles, with 5‐FU (800 mg/m^2^) administered continuously from days 1 to 5 and cisplatin (80 mg/m^2^) administered on day 1 only, every 3 weeks. DCF therapy was administered over 3 cycles, involving consecutive 5‐FU (700 mg/m^2^) administration from days 1 to 5, and on day 1 only, DOC (70 mg/m^2^) and cisplatin (70 mg/m^2^). Creatinine clearance was assessed before commencing treatment, and the therapeutic dosage was calculated using standardized criteria. Furthermore, the doses of variety of medications were adjusted or reduced in response to the toxicity observed in prior treatment cycles.

### 2.4 Patient background characteristics retrieval

All patients' clinical data were retrieved from our meticulously maintained database. Prior to and following neoadjuvant chemotherapy, assessments were conducted for height, weight, and body mass index (BMI). Preoperatively, laboratory data included the prognostic nutritional index, calculated as (10 × serum albumin [g/dl]) + (0.005 × total lymphocyte count [/mm^2^]). Clinical staging was determined in accordance with the Japanese Esophageal Society's “Japanese Classification of Esophageal Cancer” (11th edition).^11^ The same reference was used for tumor type. Smoking history was evaluated using the Brinkman index,^12^ which is calculated as the number of cigarettes smoked per day multiplied by the number of years smoked. Performance status was defined using the American Society of Anesthesiologists physical status (ASA‐PS) score.^13^ In this study, stenosis was defined as the inability to passage standard EGD.

### 2.5 Measurement and definitions of body composition

Patient body composition was assessed using CT imaging for both diagnostic purposes prior to neoadjuvant chemotherapy and to assess the impact of chemotherapy post‐neoadjuvant chemotherapy. In the present study, we quantified skeletal muscle mass, subcutaneous fat mass, and visceral fat mass with CT scans conducted pre‐ and post‐neoadjuvant chemotherapy. The timing of CT imaging was approximately every 2 months for CF and around 3 months for DCF. Skeletal muscle mass was calculated by tracing and manually measuring the total cross‐sectional area of skeletal muscle at the third lumbar vertebra level, which was then divided by the square of the patient's height to derive the skeletal muscle mass index (SMI) in cm^2^/m^2^. The cutoff values for SMI were established at 42.0 cm^2^/m^2^ in men and 38.0 cm^2^/m^2^ in women, a criterion also introduced in the Guidelines on Sarcopenia in Liver Disease (1st edition)^10^ presented by the Japan Society of Hepatology in 2016. The same CT images were used to calculate visceral and subcutaneous fat at the umbilical level, with Hounsfield units (HU) thresholds set within the range of −190 to −30 for subcutaneous adipose tissue and − 150 to −50 for visceral adipose tissue. The assessment of skeletal muscle and visceral fat masses was performed with the Volume Analyzer Synapse VINCENT 3 image analysis system from Fujifilm Medical (Tokyo, Japan), and the percentage change pre‐ and post‐neoadjuvant chemotherapy was determined using the following formulas:

$\%△\mathrm{BMI}=\left(\text{post}-\text{neoadjuvant chemotherapy}\;\mathrm{BMI}-\mathrm{pre}-\text{neoadjuvant chemotherapy}\;\mathrm{BMI}\right)/\mathrm{pre}-\text{neoadjuvant chemotherapy}\;\mathrm{BMI}\times 100.$

$\%△\mathrm{SMI}=\left(\text{post}-\text{neoadjuvant chemotherapy}\;\mathrm{SMI}-\mathrm{pre}-\text{neoadjuvant chemotherapy}\;\mathrm{SMI}\right)/\mathrm{pre}-\text{neoadjuvant chemotherapy}\;\mathrm{SMI}\times 100.$

$\%△\text{Visceral}\;\mathrm{fat}=\left(\text{visceral}\;\mathrm{fat}\;\text{after neoadjuvant chemotherapy}-\text{visceral}\;\mathrm{fat}\;\text{before neoadjuvant chemotherapy}\right)/\text{visceral}\;\mathrm{fat}\;\text{before neoadjuvant chemotherapy}\times 100.$ $\%△\text{Subcutaneous}\;\mathrm{fat}=\left(\text{subcutaneous}\;\mathrm{fat}\;\text{after neoadjuvant chemotherapy}-\text{subcutaneous}\;\mathrm{fat}\;\text{before neoadjuvant chemotherapy}\right)/\text{subcutaneous}\;\mathrm{fat}\;\text{before neoadjuvant chemotherapy}\times 100.$

Skeletal muscle mass falling below the established cutoff value was characterized as low muscle mass. For both sexes, the lower 25% were classified as low visceral fat and low subcutaneous fat groups, respectively. The group transitioning to low visceral fat and low subcutaneous fat following treatment was designated as “shift to low visceral fat” and “low subcutaneous fat.”

### 2.6 Nutritional screening

In this study, nutritional screening employed the subjective global assessment (SGA) advocated by Baker et al.^14^ This SGA is a widely used nutritional screening tool in Japan. This method has been utilized to predict the length of hospital stay for patients with digestive cancer and consists of clinical history and physical examination findings.^15^ The medical history segment consists of four items: weight loss, gastrointestinal symptoms, functional capacity, and complications. Physical examination was assessed by subcutaneous fat loss, muscle wasting, and edema. Patients were categorized into the well‐nourished, mild‐to‐moderate malnourished, and severe malnourished based on their responses to the SGA questions. The SGA is a subjective assessment of an individual's general nutritional status based on an interview, medical history, and simple physical findings by inspection and palpatory.

### 2.7 Nutritional management

No special nutritional protocols were established in this study, but oral intake was adopted as the basis for the study. Nutritional management is the same in both CF and DCF and has the following policies: if the patient's physician or staff in charge judged that the patient had difficulty eating due to stenotic symptoms from the primary disease or adverse events from chemotherapy, and that the patient's food intake was poor, the patient was managed with EN and total parenteral nutrition. Basically, there have been no cases of percutaneous endoscopic gastrostomy because the patient is scheduled for surgery in the future.

The nutritionist also performed nutritional assessment by SGA and recommended nutrition support team (NST) intervention if the patient had poor nutritional status by SGA and had poor food intake.

### 2.8 Definition of poor prognosis

In this study, we define a poor prognosis as the occurrence of mortality based on the evaluation of OS. Poor prognosis is ascertained when patients experience death during the observation period, which is a common approach in assessing the long‐term outcomes of the studied population.

### 2.9 Statistical analysis

We conducted statistical analyses with JMP version 9.0.2 software by SAS Institute in Cary, North Carolina, USA. Continuous variables were expressed as medians, means, and ranges, and differences between the medians were assessed nonparametrically using the Mann–Whitney U test. Category variables were presented as number of patients, and their distribution differences groups were examined using the χ^2^‐square test. The survival curves were estimated by the Kaplan–Meier method, while group comparisons were conducted using the log‐rank test. To identify predictors of OS, univariate, and multivariate Cox regression analyses were employed. We considered a *p*‐value of less than .05 to be statistically significant.

## 3 RESULTS

### 3.1 Patients' baseline characteristics at diagnosis

The median age of the entire patient cohort was 67 years. Of the registered patients, 178 were male and 37 were female. Among them, 91 had clinical Stage II disease, and 124 had clinical Stage III disease. Additionally, 146 patients received CF as a regiment of neoadjuvant chemotherapy, and 69 patients received DCF. After neoadjuvant chemotherapy, the median serum albumin level was 4.0 g/dL, and the prealbumin level was 26.8 mg/dL. The median BMI before neoadjuvant chemotherapy was 21.3 kg/cm^2^. Among patients who underwent standard EGD prior to neoadjuvant chemotherapy, 42 (19.5%) had stenosis (Table 1).

**TABLE 1:** Patients' baseline characteristics at diagnosis.

Patients' data	
Age, years old	67 (40–81)
Sex, *n* (%)	
Male	178 (82.8)
Female	37 (17.2)
BMI, kg/m^2^	21.3 (13.1–30.9)
Diabetes mellitus, *n* (%)	25 (11.6)
ASA‐PS, *n* (%)	
1	69 (32.1)
2	124 (57.7)
3	22 (10.2)
Cardiovascular disease, *n* (%)	98 (45.5)
Smoking, *n* (%)	185 (86.0)
Brinkman index	600 (0–2501)
Clinical staging	
Location, *n* (%)	
Ut	31 (14.4)
Mt	106 (49.3)
Lt	78 (36.3)
Typing, *n* (%)	
0	48 (22.3)
I	22 (10.2)
II	82 (38.1)
III	63 (29.4)
Clinical T (1/2/3/4a), *n* (%)	
1	48 (22.3)
2	43 (20.0)
3	121 (56.3)
4a	3 (1.4)
Clinical N (0/1/2/3), *n* (%)	
0	26 (12.1)
1	93 (43.3)
2	82 (38.1)
3	14 (6.5)
Clinical stage (II/III), *n* (%)	
II	91 (42.3)
III	124 (57.7)
NAC, *n* (%)	
CF	146 (67.9)
DCF	69 (32.1)
Pathologic data	
%FEV1	77.46 (53–106.3)
%VC	101.1 (54.7–142)
Alb, g/dl	4.0 (2.7–4.7)
Prealbumin, (mg/dl)	26.8 (10.4–43.4)

*Note*: clinical T, clinical N, and clinical stage are used in the clinical findings of the Japanese classification of esophageal cancer. Age, BMI, Brinkman index, %FEV1.0, %VC, Alb, and prealbumin are in median (range). Reference value; %FEV1> 70, %VC> 80, Alb> 3.9 g/dL, prealbumin 22.0–40.0 mg/dL.

Abbreviations: Alb, albumin; ASA‐PS, American Society of Anesthesiologists physical status; BMI, body mass index; %FEV1, %forced expiratory volume in 1 s; Lt, lower thoracic esophagus; Mt, middle thoracic esophagus; NAC, neoadjuvant chemotherapy; NST, nutrition support team; SGA, subjective global assessment; Ut, upper thoracic esophagus; %VC, %vital capacity.

Table 2 displays the group baseline characteristics of the CF and DCF. In comparison to the CF patients, the DCF patients were significantly younger (*p* < .01), had a higher percentage of male patients (*p* = .03), and had a greater incidence of pre‐treatment stenosis (*p* = .02). The prevalence of clinical Stage III was higher in the DCF group (*p* < .01). Nevertheless, there were no differences regarding the ASA‐PS score, medical history, or the presence of underlying medical conditions.

**TABLE 2:** Comparison of patient's characteristics in the CF and DCF groups.

	CF	DCF	*p* value
*N*	146	69	
Patients' data			
Age, years old^a^	66.2 ± 8.5	62.7 ± 8.8	<.01
Sex, *n*			.03
Male	115	63	
Female	31	6	
Pre BMI (kg/m^2^)^a^	21.6 ± 3.13	21.1 ± 2.71	.19
Post BMI (kg/m^2^)^a^	21.3 ± 2.9	21.5 ± 2.68	.63
DM, *n* (%)	17 (11.6)	8 (11.6)	1.00
ASA‐PS, *n*			.32
1	45	24	
2	83	41	
3	18	4	
Cardiovascular disease, *n* (%)	70 (47.9)	28 (40.6)	.38
Smoking, *n* (%)	121 (82.9)	64 (92.8)	.05
Brinkman index^a^	600 ± 494	723 ± 537	.10
Weight loss rate (pre‐treatment)^a^	2.57 ± 4.28	4.49 ± 5.09	.01
Clinical staging			
Stenosis, *n* (%)	22 (15.1)	20 (29.0)	.02
Location, *n*			.43
Ut	18	13	
Mt	74	31	
Lt	54	25	
Typing, *n*			<.01
0	44	4	
I	16	6	
II	47	35	
III	39	24	
Clinical T, *n*			<.01
1	44	4	
2	36	7	
3	66	55	
4a	0	3	
Clinical N, *n*			<.01
0	23	3	
1	74	19	
2	44	38	
3	5	9	
Clinical stage, *n*			<.01
II	80	11	
III	66	58	
Pathological data			
%FEV1^a^	77.6 ± 8.2	77.1 ± 7.7	.72
%VC^a^	102.2 ± 12.5	101.7 ± 13.1	.82
Nutritional data			
Nutritional management by the NST, *n* (%)	11 (7.53)	9 (13.0)	.21
Parenteral nutrition management, *n* (%)	7 (4.8)	4 (5.8)	.72
IVH	2	2	
Feeding tube	5	2	
SGA, *n*			.39
A	123	54	
B	14	11	
C	9	4	

Abbreviations: Alb, albumin; ASA‐PS, American Society of Anesthesiologists physical status; BMI, body mass index; CF, 5‐fluorouracil and cisplatin; DCF, docetaxel, cisplatin, and 5‐fluorouracil; DF, %FEV1, %forced expiratory volume in 1 s; Lt, lower thoracic esophagus; Mt, middle thoracic esophagus; NAC, neoadjuvant chemotherapy; NST, nutrition support team; SGA, subjective global assessment; Ut, upper thoracic esophagus; %VC, %vital capacity.

^a^ Mean ± standard deviation.

When comparing treatments (Table 3), Grade 3 or above chemotherapy‐related adverse events were significantly more frequent in the DCF group than in the CF group across all categories: fatigue (*p* < .01), anorexia (*p* = .01), neutropenia (*p* < .01), and diarrhea (*p* < .01). However, neoadjuvant chemotherapy was discontinued in 16 (10.9%) patients in the CF group and 4 (5.8%) patients in the DCF group, with no significant difference between them. Nutritional support was provided by NST with 11 (7.5%) patients in the CF group and 9 (13.0%) patients in the DCF group during neoadjuvant chemotherapy, significantly different between both groups.

**TABLE 3:** Comparison of CF and DCF regarding treatment.

	CF	DCF	*p* value
*N*	146	69	
Discontinuation of chemotherapy, *n* (%)	16 (10.9)	4 (5.8)	.31
Adverse events of chemotherapy ≧Grade3			
Fatigue, *n* (%)	6 (4.1)	9 (13.0)	<.01
Anorexia, *n* (%)	9 (6.2)	9 (13.0)	.01
Neutropenia, *n* (%)	33 (22.6)	50 (72.5)	<.01
Diarrhea, *n* (%)	1 (0.7)	3 (4.3)	<.01
Response evaluation, *n*			.13
CR	4	5	
PR	100	52	
SD	30	7	
PD	12	5	
Post‐NAC hematological examination			
Alb, g/dl^a^	4.02 ± 0.32	3.79 ± 0.35	<.01
Hb,g/dl^a^	12.1 ± 1.35	11.3 ± 1.1	<.01
ChE, IU/L^a^	291 ± 60.1	276.4 ± 57.0	.09
Prealbumin, mg/dl^a^	26.8 ± 5.2	26.1 ± 5.2	.35
CRP, mg/dl^a^	0.25 ± 0.49	0.22 ± 0.63	.72
Lymphocytes, /mm^3a^	1566.8 ± 525.9	1555.1 ± 493.7	.88

*Note*: Response evaluation is based on response evaluation criteria in solid tumors. Reference value; Hb 12‐18 g/dL, ChE 100‐240 IU/L, CRP 0–0.3 mg/dL, lymphocytes >1500/mm^3^, and PNI 50–60.

Abbreviations: Alb, albumin; CF, 5‐fluorouracil and cisplatin; ChE, cholinesterase; CR, complete response; CRP, C‐reactive protein; DCF, docetaxel, cisplatin, and 5‐fluorouracil; Hb, hemoglobin; PD, progressive disease; PNI, prognostic nutritional index; PR, partial response; SD, stable disease.

^a^ Mean ± standard deviation.

### 3.2 Body composition changes before and after neoadjuvant chemotherapy

Tables 4 and 5 provide an overview of the body composition pre‐ and post‐neoadjuvant chemotherapy in both groups. The changes in body composition pre‐ and post‐neoadjuvant chemotherapy are detailed in Table 6. It is worth noting that before neoadjuvant chemotherapy, 130 patients (89.1%) in the CF group and 63 patients (91.3%) in the DCF group exhibited low muscle mass, with no statistically significant distinction between both groups. After neoadjuvant chemotherapy, 140 patients (95.9%) in the CF group and 62 patients (89.9%) in the DCF group had low muscle mass, with no significant difference observed. There were no significant differences regarding visceral fat mass before and after neoadjuvant chemotherapy. However, the subcutaneous fat mass was notably lower in the DCF group both pre‐ and post‐neoadjuvant chemotherapy. Nevertheless, there were no statistically significant differences between both groups concerning lower subcutaneous fat.

**TABLE 4:** Comparison of body composition of CF and DCF before NAC.

	CF	DCF	*p* value
*N*	146	69	
SMI, cm^2^/m^2^ ^a^	40.4 ± 8.3	41.5 ± 7.6	.36
Subcutaneous fat, cm^2^ ^a^	97.8 ± 52.1	79.1 ± 42.9	<.01
SATI, cm^2^/m^2^	37.2 ± 21.3	28.5 ± 15.8	<.01
Visceral fat, cm^2^ ^a^	105.5 ± 55.7	92.1 ± 50.1	.12
VATI, cm^2^/m^2^	39.1 ± 20.7	32.6 ± 18.2	<.03
Low subcutaneous fat, *n* (%)	33 (22.6)	19 (27.5)	.49
Low visceral fat, *n* (%)	33 (2.6)	19 (27.5)	.49
Low muscle mass, *n* (%)	130 (89.1)	63 (91.3)	.81

Abbreviations: CF, 5‐fluorouracil and cisplatin; DCF, docetaxel, cisplatin, and 5‐fluorouracil; NAC, neoadjuvant chemotherapy; SATI, subcutaneous adipose tissue index; SMI, skeletal mass index; VATI, visceral adipose tissue index.

^a^ Mean ± standard deviation.

**TABLE 5:** Comparison of body composition of CF and DCF after NAC.

	CF	DCF	*p* value
*N*	146	69	
SMI, cm^2^/m^2^ ^a^	38.4 ± 7.5	40.3 ± 7.2	.08
Subcutaneous fat, cm^2^ ^a^	93.1 ± 49.4	78.6 ± 40.3	.03
SATI, cm^2^/m^2^	35.2 ± 20.1	28.2 ± 14.6	.01
Visceral fat, cm^2^ ^a^	97.2 ± 50.3	85.7 ± 39.9	.10
VATI, cm^2^/m^2^	35.4 ± 19.0	30.6 ± 13.9	.06
Low subcutaneous fat, *n* (%)	32 (21.9)	21 (30.43)	.18
Low visceral fat, *n* (%)	41 (28.1)	20 (28.9)	1.00
Low muscle mass, *n* (%)	140 (95.9)	62 (89.9)	.12

Abbreviations: CF, 5‐fluorouracil and cisplatin; DCF, docetaxel, cisplatin, and 5‐fluorouracil; NAC, neoadjuvant chemotherapy; SATI, subcutaneous adipose tissue index; SMI, skeletal mass index; VATI, visceral adipose tissue index.

^a^ Mean ± standard deviation.

**TABLE 6:** Comparison of body composition changes before and after NAC for CF and DCF.

	CF	DCF	*p* value
*N*	146	69	
%△BMI^a^	−1.4 ± 4.8	2.3 ± 5.5	<.01
%△SMI^a^	−4.4 ± 7.8	−2.4 ± 8.3	.08
%△visceral fat^a^	−2.1 ± 33.8	5.9 ± 43.8	.14
%△subcutaneous fat^a^	−1.2 ± 30.5	17.9 ± 103.2	.04
Shift to low visceral fat, *n* (%)	14 (9.6)	5 (7.3)	.79
Shift to low subcutaneous fat, *n* (%)	7 (4.8)	4 (5.8)	.74

Abbreviations: BMI, body mass index; CF, 5‐fluorouracil and cisplatin; DCF, docetaxel, cisplatin, and 5‐fluorouracil; NAC, neoadjuvant chemotherapy; SMI, skeletal mass index.

^a^ Mean ± standard deviation.

Furthermore, the SMI and visceral fat mass in both groups was consistent throughout the neoadjuvant chemotherapy treatment. The only significant change was a decrease in BMI in the CF group. The numbers of patients who had sarcopenia, exhibited decreased visceral fat, or had decreased subcutaneous fat were not significantly different between the two groups during the treatment period.

### 3.3 Prognostic factors

**FIGURE 1:**
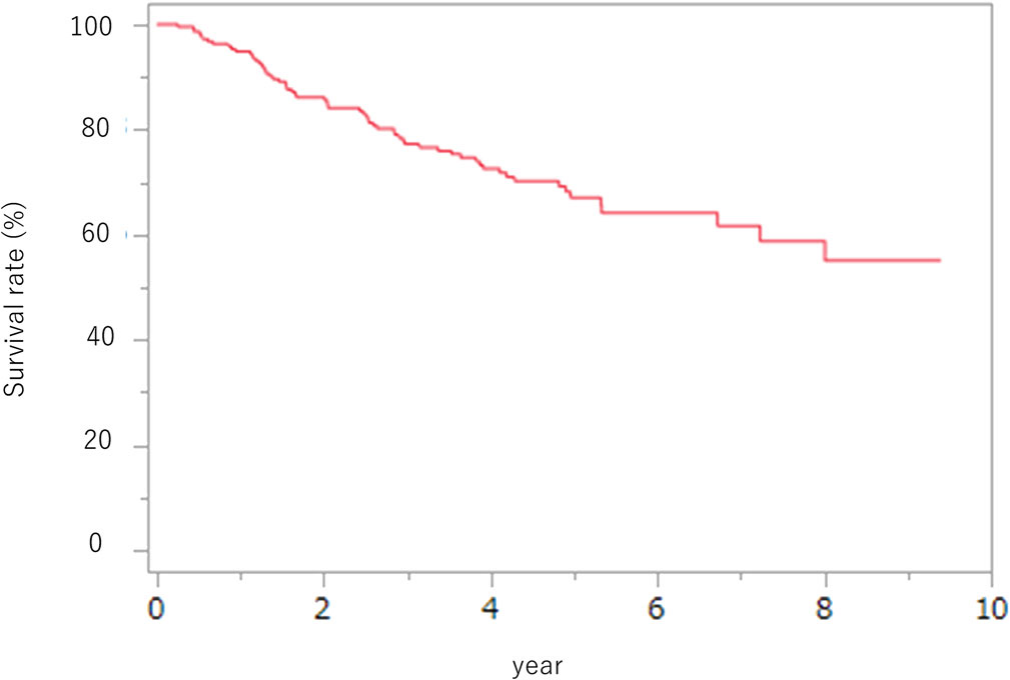
Survival curves for all patients in this study. The median overall survival of patients was not reached; the 5‐year survival rate was 67.1%.

The median OS of patients was not reached; the 5‐year survival rate was 67.1% (Figure 1). In Table 7, we present the key prognostic factors for patients who underwent surgery following neoadjuvant chemotherapy for thoracic esophageal cancer. The univariate analysis considered variables such as clinical Stage III disease, post‐neoadjuvant chemotherapy low muscle mass, a transition to low visceral fat, and response evaluation (SD/PR). In the multivariate analysis, it was established that clinical Stage III disease, a shift to low visceral fat, and the response evaluation (SD/PD) were the notable prognostic factors. It is noteworthy that our study did not find variations in neoadjuvant chemotherapy to be significant prognostic factors.

**TABLE 7:** Poor prognostic factors for resectable advanced‐stage esophageal cancer.

		Univariate analysis		Multivariate analysis	
		HR (range)	*p* value	HR (range)	*p* value
Sex	Male	1.5 (0.74–3.31)	0.29		
	Female	1			
Age	>60	1.79 (095–3.73)	0.07		
	≦60	1			
ASA‐PS	≧2	1.05 (0.63–1.81)	0.84		
	1	1			
cStage	III	2.06 (1.21–3.67)	<0.01	1.84 (1.06–3.18)	.02
	II			1	
Neoadjuvant chemotherapy	CF	1.3 (0.76–2.31)	0.33		
	DCF	1			
Low muscle mass (post)	Present	5.03 (1.11–89.0)	0.03	3.34 (0.45–24.3)	.23
	Absent	1		1	
Response evaluation	SD/PD	2.81 (1.69–4.69)	<0.01	2.47 (1.28–4.76)	<.01
	CR/PR	1		1	
Low muscle mass (pre)	Present	1.57 (0.69–4.51)	0.3		
	Absent	1			
Low visceral fat (pre)	Present	1.42 (0.82–2.43)	0.21		
	Absent	1			
Low subcutaneous fat (pre)	Present	1.65 (0.94–2.88)	0.08		
	Absent	1			
Shift to low visceral fat	Present	2.85 (1.41–5.26)	<0.01	2.46 (1.27–4.75)	<.01
	Absent	1		1	
Shift to low subcutaneous fat	Present	1.51 (0.60–3.78)	0.37		
	Absent	1			

Abbreviations: ASA‐PS, American Society of Anesthesiologists physical status; CR, complete response; HR, hazard ratio; PD, progressive disease; PR, partial response; SD, stable disease.

## 4 DISCUSSION

In our study, a substantial portion of patients exhibited low muscle mass before initiating treatment. However, it is important to note that the prevalence of low muscle mass did not differ significantly between the CF and DCF groups before treatment, and there were no significant variations observed in the alterations of skeletal muscle mass during neoadjuvant chemotherapy or incidences of low muscle mass post‐treatment. A similar pattern emerged in the assessment of visceral fat mass, with no significant disparities observed between the two groups pre‐ and post‐treatment. Furthermore, the rate of change did not exhibit a substantial difference. Consequently, when examining body composition during treatment, there were no statistically significant differences between the CF and DCF.

However, over the long term, the transition to low visceral fat during this treatment phase was found to have a notable impact on patient prognosis. This outcome underscores the importance of implementing nutritional management during this critical period.

In prior studies,^9^, ^16^ we previously reported changes of body composition that occurred in patients undergoing neoadjuvant chemotherapy. These investigations have highlighted that change in body composition, especially skeletal muscle mass, have a significant impact on the occurrence of perioperative complications within 2–3 months post‐neoadjuvant chemotherapy treatment and their prognosis. Consequently, our examination delves into the necessity for nutritional intervention with regard to body composition. The significantly higher occurrence of sarcopenia among patients diagnosed as thoracic esophageal cancer prior to neoadjuvant chemotherapy highlights the importance of nutritional management and rehabilitation interventions prior to the initiation of neoadjuvant chemotherapy.

Besides clinical stage, low muscle mass, response evaluation, and the shift to low visceral fat emerged as predictors of prognosis in the univariate analysis. In the multivariate analysis, it was the shift to low visceral fat that significantly predicted patient outcomes. This outcome aligns with prior reports,^17^ reinforcing that in clinical studies,^18^, ^19^ assessing visceral fat as opposed to SMI serves as a more robust prognostic indicator. This underscore the necessity for ongoing nutritional intervention so far as to prevent further worsening of body composition throughout the course of treatment.

Furthermore, it is imperative to emphasize the importance of continuous nutritional support while concurrently integrating rehabilitation and nutritional management, as indicated by previous research. On the contrary, the DCF group showed a significant decrease subcutaneous fat mass, which remained significantly lower than the CF group following their treatment. The DCF group exhibited a substantial increase in rate of change throughout neoadjuvant chemotherapy treatment in contrast to the CF group. It is essential to underscore that subcutaneous adipose tissue primarily undergoes metabolism during periods of reduced physical activity and limited nutrient intake. Visceral fat typically exhibits higher sensitivity to weight loss compared to subcutaneous fat.^20^, ^21^ Nevertheless, it is crucial to consider that Hall et al.^22^ reported a deviation from the preference for visceral fat over subcutaneous fat when substantial loss of weight occurs. These imply that the DCF group might have undergone more pronounced loss of weight prior to treatment, likely because of symptoms related to a sense of fullness and insufficient food intake. Furthermore, a prior report^23^ observed a decrease in subcutaneous fat before the reduction of visceral fat and muscle mass. It is plausible that, in esophageal cancer patients, changes in food intake, in addition to the metabolic shifts linked to malignancy before treatment, could have contributed to a further decline in subcutaneous fat. Interestingly, the DCF group exhibited a significant increase in subcutaneous fat, despite neoadjuvant chemotherapy treatment being administered for a relatively short duration. With a state of positive energy balance, the adipocytes expand both size and number in order to meet the demand for storage of fat, and subcutaneous fat is the primary reservoir for adipose tissue stored. When the storage capacity of subcutaneous fat is exceeded, fat accumulation occurs in visceral fat and ectopic tissues.^24^ Consistent with previous studies reporting that effective treatment led to enhanced management of dysphagia and nutritional status,^25^, ^26^ anorexia was more prevalent in the DCF group due to chemotherapy‐related adverse events in this study. Nevertheless, this anorexia may have been transient and potentially led to an increase in patients' food intake, shifting the energy balance in a positive direction.

Malnutrition is a significant concern for esophageal cancer patients and is closely linked to a poorer prognosis.^27^ Research indicates that 60%–80% of these patients are malnourished at the time of diagnosis, with 36% experiencing severe malnutrition during surgery.^28^ Despite this, strategies that are effective in preserving or enhancing the nutrient levels of patients who undergo neoadjuvant chemotherapy remain unclear.

EN is recommended in cases of severe malnutrition or when dysphagia hinders sufficient oral intake, including liquid consumption, during the initial evaluation. Notably, it has been reported^29^ that factors influencing nutritional status during neoadjuvant chemotherapy include pre‐neoadjuvant chemotherapy albumin levels, BMI, and the utilization of EN. However, approximately 27.6% of patients may not tolerate EN effectively due to discomfort during the insertion of the feeding tube and tube blockages.

Since certain patients may still be able to consume food orally as a result of treatment effects, the necessity for nutritional management throughout neoadjuvant chemotherapy may vary from case to case and should be the collaborated with the NST. Early nutritional intervention can reduce the rate of weight loss and shorten hospital stays.^30^ These interventions hold promise for enhancing patient contentment and quality of life while safeguarding nutrient levels.

This study has several limitations that need to be acknowledged. First, it was a single‐center, retrospective study and included only patients who had computed tomography pre‐ and post‐neoadjuvant chemotherapy may have led to a selection bias. Additionally, as it relied on chart surveys, it may not have fully captured all the events and details that transpired during neoadjuvant chemotherapy treatment for esophageal cancer.

Furthermore, it is essential to note that this study was not designed as a nutrient intervention study. As a result, it could not definitively establish any direct impact of nutritional management throughout neoadjuvant chemotherapy on improving body composition and perioperative outcomes. Therefore, further research should aim to define criteria for the most efficacious nutritional interventions and to identify the optimal nutritional supplementation to enhance body composition and clinical practice outcomes in esophageal cancer patients undergoing neoadjuvant chemotherapy. This will ideally involve prospective multicenter studies to provide more robust and generalizable insights.

## 5 CONCLUSION

In patients with clinical Stage II/III advanced‐stage esophageal squamous cell carcinoma, body composition remained largely unchanged after neoadjuvant chemotherapy, regardless of treatment regimen. Other than SD or PD in treatment effect/clinical Stage III, the transition to low visceral fat during neoadjuvant chemotherapy treatment was a prognostic factor in these patients; prospective studies are essential to establish the best approach to nutritional management in the future, with the possible need for sustained nutritional support during neoadjuvant chemotherapy treatment.

## AUTHOR CONTRIBUTIONS

**Sachiyo Onishi:** Conceptualization (lead); formal analysis (lead); investigation (lead); methodology (lead); software (lead); supervision (equal); validation (lead); visualization (lead); writing – original draft (lead); writing – review and editing (lead). **Masahiro Tajika:** Conceptualization (supporting); supervision (lead); writing – review and editing (supporting). **Tsutomu Tanaka:** Conceptualization (equal). **Keisaku Yamada:** Conceptualization (equal). **Yoshitaka Inaba:** Conceptualization (equal). **Tetsuya Abe:** Conceptualization (equal). **Kei Muro:** Conceptualization (equal). **Masahito Shimizu:** Conceptualization (equal). **Yasumasa Niwa:** Conceptualization (equal); supervision (lead); writing – review and editing (supporting).

## FUNDING INFORMATION

This study did not receive funding from any public, commercial, or non‐profit organizations.

## CONFLICT OF INTEREST STATEMENT

Dr. Muro received funding from Eisai, Amgen, Taiho Pharmaceutical, Daiichi Sankyo, Sanofi, Astellas Pharma, Ono Pharmaceutical, Novartis Pharma, and Pfizer. He also received honoraria (lecture fees) from Ono Pharmaceutical, MSD, Takeda Pharmaceutical, Eli Lilly, Taiho Pharmaceutical, Daiichi Sankyo, and Bristol‐Myers Squibb. Other authors declare no competing interests.

## Supporting information


**Table S1.** A small summary table that shows only the significant results from this study.

## Data Availability

The data that support the findings of this study are available from the corresponding author upon reasonable request.
