# Supplementary material for: Prognostic impact of shift to low visceral fat mass after neoadjuvant chemotherapy in patients with esophageal cancer
Source: Cancer Rep (Hoboken). 2024 Aug 19;7(8):e2084. doi: 10.1002/cnr2.2084 (PMC11331502; doi:10.1002/cnr2.2084)
Supplement: Supplementary file 1 — Table S1. A small summary table that shows only the significant results from this study. [file CNR2-7-e2084-s001.docx]

**Supplement1 a small summary table that shows only the significant results from this study.**

|  | CF | DCF | *p* value |
| --- | --- | --- | --- |
| N | 146 | 69 |  |
| **Adverse events of chemotherapy ≧Grade3** |  |  |  |
| Fatigue, n (%) | 6 (4.1) | 9 (13.0) | <0.01 |
| Anorexia, n (%) | 9 (6.2) | 9 (13.0) | 0.01 |
| Neutropenia, n (%) | 33 (22.6) | 50 (72.5) | <0.01 |
| Diarrhea, n (%) | 1 (0.7) | 3 (4.3) | <0.01 |
| **Post-** **neoadjuvant chemotherapy hematological examination** |  |  |  |
| Alb, g/dl * | 4.02 ± 0.32 | 3.79 ± 0.35 | <0.01 |
| Hb,g/dl * | 12.1 ± 1.35 | 11.3 ± 1.1 | <0.01 |
| **Body composition before neoadjuvant chemotherapy** |  |  |  |
| subcutaneous fat, cm^2^ * | 97.8 ± 52.1 | 79.1 ± 42.9 | <0.01 |
| SATI, cm^2^/m^2^* | 37.2 ± 21.3 | 28.5 ± 15.8 | <0.01 |
| VATI, cm^2^/m^2^ * | 39.1 ± 20.7 | 32.6 ± 18.2 | <0.03 |
| **Body composition after neoadjuvant chemotherapy** |  |  |  |
| subcutaneous fat, cm^2^ * | 93.1 ± 49.4 | 78.6 ± 40.3 | 0.03 |
| SATI, cm^2^/m^2^ * | 35.2 ± 20.1 | 28.2 ± 14.6 | 0.01 |
| **Body composition change before and after neoadjuvant chemotherapy** |  |  |  |
| %△BMI * | −1.4 ± 4.8 | 2.3 ± 5.5 | <0.01 |
| %△subcutaneous fat * | −1.2 ± 30.5 | 17.9 ± 103.2 | 0.04 |

NOTES: *Mean ± standard deviation, Response evaluation is based on Response Evaluation Criteria in Solid Tumors (RECIST).

Abbreviations: Alb, albumin; Hb, Hemoglobin; SATI, subcutaneous adipose tissue index; VATI, visceral adipose tissue index; BMI, Body mass index
